# Mapping the role of digital health technologies in the case detection, management, and treatment outcomes of neglected tropical diseases: a scoping review

**DOI:** 10.1186/s41182-021-00307-1

**Published:** 2021-02-22

**Authors:** Binyam Tilahun, Kassahun Dessie Gashu, Zeleke Abebaw Mekonnen, Berhanu Fikadie Endehabtu, Dessie Abebaw Angaw

**Affiliations:** 1 Department of Health Informatics, Institute of Public Health, College of Medicine and Health Sciences, University of Gondar, Gondar, Ethiopia; 2 Health System Directorate, Ministry of Health, Addis Ababa, Ethiopia; 3 Department of Epidemiology and Biostatistics, Institute of Public Health, College of Medicine and Health Science, University of Gondar, Gondar, Ethiopia

**Keywords:** Neglected tropical diseases, Digital health technologies, Case detection, Disease management, Treatment outcome

## Abstract

**Background:**

Neglected tropical diseases (NTDs) are a diverse group of communicable diseases that principally impact the world’s poorest people. The use of digital health technologies is an emerging and promising way to improve disease prevention, diagnosis, case detection, treatment delivery, and patient follow-up and facilitating health facility appointments thereby improving health outcomes. While the growing implementation of digital health technologies is evident, there is a lack of comprehensive evidence on the impact of digital health technologies in the control of NTDs. The main objective of this review was to map different pieces of evidence on the use of digital health technologies for case detection, management, and treatment outcome of the neglected tropical diseases.

**Methods:**

We conducted a scoping review guided by the Joanna Briggs Institute guidelines. The studies were searched using electronic databases like MEDLINE (PubMed), Science Direct, Cochrane Library, and manual searching engines. Two authors extracted the data and compared the results. Discrepancies were resolved by discussion or the third reviewer made the decision. We produced the distribution of geographical locations, residents (setting), types of publications, and digital health technologies used on neglected tropical diseases using tables and graphs.

**Findings:**

A total of 996 potentially relevant studies were generated from the initial search, and six studies were found to satisfy all the inclusion criteria and included in the final review. The review found that telehealth, eHealth, mHealth, telemedicine, and electronic health record were used digital health technologies to assess their impact on case detection, disease management, and treatment outcome of neglected tropical diseases. Mobile health was a feasible digital health technology for lymphatic filariasis patient identification and mHealth, eHealth, and electronic health records found to improve the service access, outcomes, and monitoring of visceral leishmaniasis at the community health system.

**Conclusion:**

The scoping review identified that there were limited studies to see the impact of digital health technologies on case detection, management, and treatment outcomes for neglected tropical diseases. We also found that digital health technologies like ehealth, electronic medical linkage, telemed, and telehealth were practicable for patient identification, for treatment and diagnosis through contact with health professionals and teleconsultation, and support in improving health service delivery at the community-health system for managing the disease in both rural and urban settings.

**Supplementary Information:**

The online version contains supplementary material available at 10.1186/s41182-021-00307-1.

## Background

The neglected tropical diseases (NTDs) are a diverse group of communicable diseases that prevail in tropical and subtropical conditions in 149 countries that causes substantial illness for more than 1.5 billion of the poorest, most marginalized communities worldwide with devastating health, social, and economic consequences [1–3]. The most common neglected tropical diseases are Protozoan infections (human African trypanosomiasis, Chagas disease, and leishmaniasis), helminthic infections (ascariasis, trichuriasis, schistosmiasis, lymphatic filariasis, onchocerchiasis, and dracunculiasis), bacterial infections (Buruli ulcer, leprosy, and trachoma), and viral infection (rabies and dengue) with high endemicity in the developing countries [4–6].

According to a 2010 WHO report, neglected tropical diseases have traditionally ranked low on national and international health agendas and cause massive but hidden and silent suffering, and frequently kill the poor people [7]. NTD has many consequences like impairs physical and cognitive development, contribute to mother and child illness and death, limit productivity, and entire communities become mired in poverty as disabled and unemployed people struggle to afford food and basic services, including healthcare [8–12].

According to the World Health Organization (WHO) road map for neglected tropical diseases 2021–2030, lack of timely access to affordable treatment leaves hundreds of millions severely disabled, disfigured, or debilitated, often resulting in social exclusion, stigmatization, and discrimination [3]. Even though morbidity and mortality due to NTDs were underestimated as long-term disabilities [4, 13–15], the combined burden of disease due to NTDs has been estimated at 56.6 DALYs, compared with malaria at 46.5 and tuberculosis (TB) at 34.7 [4, 7].

The World Health Organization (WHO) recommends five public-health strategies for the prevention and control of NTDs: preventive chemotherapy; intensified case-management; vector control; the provision of safe water, sanitation, and hygiene; and veterinary public health [7]. Although strategies to control and eliminate NTDs primarily for human African trypanosomiasis, leishmaniasis, soil-transmitted helimintiasis (STH), Chagas disease, trachoma, schistosmiasis, and onchocerchiasis are available, ultimately, success will almost certainly depend on access to new and cost-effective products for improved control [1].

Currently WHO recommended for low and middle-income countries to develop non-laboratory-based diagnosis and controlling policies with low-cost ways to prevent, diagnose, and treat the diseases [16]. Additionally, the use of electronic technologies like mobile phone message/reminder, ehealth, mhealth, and medical health records facilitates education and awareness, remote data collection, remote monitoring, communication and training of healthcare providers, monitoring of disease and epidemic outbreaks, and diagnostic and treatment support [16–18].

Digital health technologies are devices used for computing platforms, facilitate connectivity (create network) and sensors for healthcare and related issues and mostly used by healthcare providers [19]. It is an emerging and promising way to improve disease prevention, diagnosis, treatment compliance, medication adherence, and honoring clinic appointments thereby enhancing health outcomes [20, 21]. A report from the agency for healthcare research and quality revealed that incorporating electronic technology in health for disease management efforts can yield significant cost savings for patients and providers [22, 23]. A review study in 2015 on the use of Telemedicine technologies in the management of infectious diseases indicated that patients with infectious and non-infectious diseases who are receiving telemedicine care report feeling more satisfied with and more involved in their care. Furthermore, patients claimed that attending remote appointments through telemedicine saved their time, diminished the distance traveled, and reduced missed workdays [24, 25].

While the growing popularity of digital health technologies is evident, and promoters are eager to demonstrate their effect on NTDs diagnosis and case finding and detection, disease management, and treatment outcome impact may be overlooked in low- and middle-income countries; people may have poor access to quality healthcare due to poor road networks, long-distance travel, lack of trained or skilled health professionals, lack of health facilities, and ignoring the disease [26, 27]. As a result of this, challenges and the potential importance of digital health technologies for improving access for healthcare; the technology could be applied on NTDs detection, management, and treatment outcome to people living hard to reach areas. We performed scoping review other than systematic review due to not miss many papers, for instance, expertise view and case report studies. In systematic review, quality of the study could be assessed weather the study is eligible or not to be incorporated in the review; whereas in scoping review, assessing the quality of the study is optional [28]. This review builds the evidence base of digital health technologies by updating previous studies and assessing a broad range of finding from usability to impact on outcomes of neglected tropical diseases. Therefore, this review aims to map evidence of digital health technologies on neglected tropical diseases detection, management, and treatment outcome of the diseases.

## Methods

Our review aimed to map the available evidence on the role of digital health technologies like electronics health record, eHealth, electronic medical records, electronic community linkage, mHealth for case detection and case finding, loss to follow-up control, and disease management of NTDs. This review was conducted within the Reporting Items for Systematic Reviews and Meta-Analyses extension for Scoping Reviews (PRISMA-ScR) checklist [29] and it was guided by Joanna Briggs Institute (JBI) scoping review guidance [28].

### The scope review research question

We used a Population, Concept, and Context (PCC) framework developed by the Joanna Briggs Institute to determine the eligibility of our primary research question [28]. The following is the primary research question: what are the available pieces of evidence of digital health technologies applied for case detection, disease management, and treatment output for neglected tropical diseases in the world?

### Eligibility criteria

Studies were included based on the following criteria: (1) Studies that reported evidence on the application of digital health technologies for one of the neglected tropical disease affected patients used for treatment support, (2) studies that report the application of one of the digital health technologies either for disease management or lost to follow-up control among one of the NTD patients, (3) articles that report a piece of evidence on the application of one of the digital health technologies for case detection, management, and treatment output, (4) pieces of evidence of digital health technologies on treatment support and lost to follow-up control from systematic reviews, meta-analyses, letters. Whereas, articles reported only evidence on the importance of digital health technologies were excluded.

### Data sources and searching strategies

The studies were searched using electronic databases like MEDLINE (PubMed), CINAHL with full-text via EBSCOhost, and green FILE; Science Direct; Cochrane Library; and Google Scholar advanced databases. Other data sources like Hinari and Google were used to search for studies. In this electronic database search, Medical Subject Headings (MeSH) were used for database searching and Boolean terms (AND/OR) were used to separate our keywords. The following keywords were used: “neglected tropical disease,” leishmaniasis, Schistosomiasis, Elephantiasis, trachoma, Dracunculiasis, “Soil-transmitted Helminths,” Onchocerciasis, “Chagas Disease,” dengue fever,” “Human African Trypanosomiasis,” Telemedicine, telemed, mobile health, Telehealth, eHealth, mHealth, “Computerized Medical Record*,” “Electronic Health Record,” “Electronic Medical Record” (medical record, computerize), “Record Linkage,” Medical, “treatment outcome,” “Patient-Relevant Outcome,” “Rehabilitation Outcome,” “Treatment Effectiveness,” “Treatment Efficacy,” lost to follow-up, “Dis management,” “case finding,” “case detection.” Additional relevant articles were identified by searching the reference lists of full-text articles studies published from conception to the last searched date of August 02, 2020, were included (Additional file 1).

### Study selection and reliability

We performed initial searches by two review authors with extensive experience in systematic reviews. Screening of titles, abstracts, and full texts was conducted independently by two review authors (DA and KD). A disagreement regarding the decision against the inclusion of articles between the two reviewers was resolved by consensus or the third reviewer (BF).

A second reviewer (KD) was blinded to the primary reviewer’s (DA) decision for checking articles selection and data extraction. Any differences of opinion were discussed; otherwise, a third reviewer (BF) was available to arbitrate any issues that remained unresolved.

### Data charting (extraction of the results)

Two authors (DA and ZA) extract the data and compared the results. Discrepancies were resolved by discussion or the third reviewer made the decision. We extracted the following data from each study: author name, year, country, types of digital health technology use, purpose of applying digital health technology (case detection and/or finding disease management and treatment support), study population, study design, types of neglected tropical diseases, and geographical setting (urban/rural) (Additional file 2 and Table 1).

**Table 1:** Summary of the main features of the 6 studies included in the scoping review on the applications of electronic technologies for treatment outcome, case detection, and disease management (2012-2018)

Variable	Frequency (%)
**Geographical location**	
India	1 (16.7)
Ghana	1 (16.7)
Tanzania	2 (33.3)
Both (Malawi and Ghana)	1 (16.7)
At Africa level (review)	1 (16.7)
**Resident**	
Rural	2 (33.3)
Urban	1 (16.7)
Both	1 (16.7)
Not specific	2 (33.3)
**Types of publication**	
Expert review	2 (33.3)
Original article	3 (50)
Thesis (unpublished)	1 (16.7)

### Collating, summarizing, and reporting

Based on the methodological framework for scoping reviews [30], we were able to present our narrative account of findings in two ways. First, attention was given to the basic numerical analysis of the extent, and distribution of the studies included in the review. We produced the distribution of geographical locations, residents (setting), types of publications, and electronic technologies used on neglected tropical diseases using tables and graphs. Second, the study findings from the existing literature were presented using thematic content analysis. Our narrative literature was then structured around the themes derived from the study results or outcomes. The themes that emerged from the study were types of electronic technologies used, treatment outcome, case identification/detection, disease management, and disease diagnosis of neglected tropical diseases.

## Results

A total of nine hundred ninety-six potentially relevant studies (790 from PubMed, 96 from Google scholar (advanced), 68 from science direct, 32 from Google,10 from CINAHL, and Cochrane library (0) were generated from the initial search. After duplicates were excluded, 890 studies remained. Then, we excluded most of these potentially relevant papers (848—95%) based on the title (*n*=780) and abstract (*n*=68) review. Overall, 42 studies were eligible for full-text screening.

**Fig. 1:**
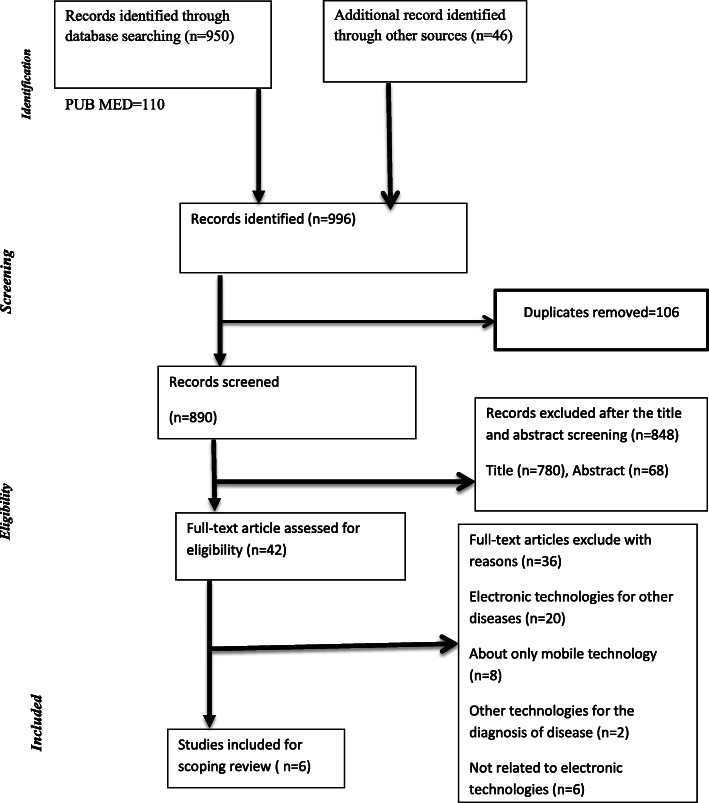
Flow diagram for the scoping review process adapted from the PRISMA statement by Moher and colleagues (2009)

Afterward, 36 studies were excluded due to electronic technologies used for another disease (*n*=20), studies deal only about mobile technology (like developing mobile technology and deal on mobile web and devices) (*n*=8), other technologies for the diagnosis of disease such as radiology, ultrasonography, and computed axial tomography (*n*=2), and not related to the electronic technologies, for instance, the impact of golf on health, impact of NTDs on socioeconomic, the burden of NTDs, and inadequate health intervention for neglected tropical diseases (*n*=6) (Fig. 1).

### Characteristics of included studies

In this review, six studies were originated from India [31], Tanzania [32, 33], Ghana [34, 35], and Malawi [34], and the review was comprised of one cross-sectional study [33], one follow-up study [35], one qualitative study [32], one case study [34], and two from expert reviews [31, 36]. Out of the six studies, two studies were from the rural setting [34, 35], one study was from urban [33], one study was from both rural and urban [32], and two studies were the setting is not specified [31, 36] , because they were not primary studies (Table 1).

**Fig. 2:**
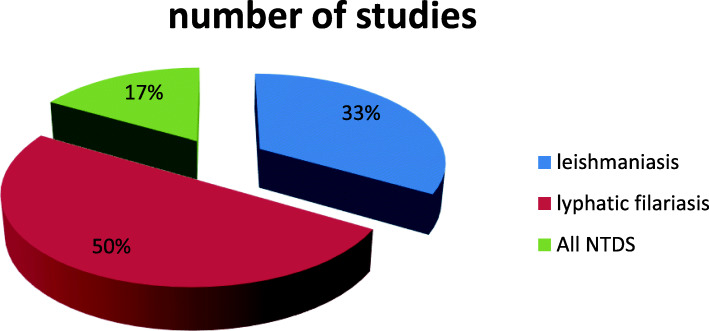
Distribution of included studies by neglected tropical diseases using electronic technologies for treatment support, disease management, case finding, and case detection

Out of the six studies, two studies were on leishmaniasis [31, 36], three studies were on lymphatic filariasis [33–35], and one was on multiple NTDs (leishmaniasis, lymphatic filariasis, Chagas disease, and dengue fever) [32] (Fig. 2 and Table 2).

**Table 2:** Summary of extracted data on the purpose of electronic technology use and their main outcome

Studies	Types NTDs	Types of electronic technology use	Primary purpose/aim	Main finding
Tambo et al.	Leishmaniasis	mHealth, eHealth, and electronic health records	**Disease management and treatment outcome** ✓ The use of digital public health technology applications in understanding and data mapping models for health and environment decisions, support in improving health service access, and uptake in Africa at the community health system	[36]
Bhunia et al.	Leishmaniasis	telehealth	**For diagnosis and treatment and vector control** ✓ To provide a good opportunity for the patients to have access to remote hospitals for real-time evaluation by specialists, via either video or audio, using Doppler and webcams ✓ To integrate multifaceted intervention, prevention, assessment, and treatment to achieve improved personnel as well as local, national, and international community health	Improved medical care to remote rural areas, including many other activities like public awareness, changing personal behavior, early diagnosis and complete treatment, integrated vector management, and vector surveillance; social mobilization and building partnerships
Madon et al.	For all NTDs	Mobil phone (SMS)	**Control of NTDs** (quantifying drug need, prioritizing disease at the local level, upward information, and facilitating decision) ✓ To test the feasibility of a mobile phone-based management information system (MIS) using frontline health workers to capture data at the point of source for control of NTDs	✓ Increase the efficiency of routine work boosting the motivation and self-esteem of village health worker ✓ Increased the flow of communication with district-level health official; increase the overall success of the NTD control program in the country
Vroom et al.	Lymphatic filariasis	mHealth	**Disease management and disease control**	The use of mobile phones does not guarantee improved data accuracy. However, the type of mobile technology used may aid in improving data completeness
Mwingira et al.	Lymphatic filariasis	mHealth	**Case detection** ✓ Lymphatic filariasis patient identification	The approach is a feasible framework that could be used in other large urban environments in the LF endemic areas
Stanton et al.	Lymphatic filariasis	Mobile message (SMS)	**Case management/disease management** ✓ To develop and test an SMS tool (measure SMS) which enables trained community-based health workers to report basic information on all cases they identified	✓ Enabled national programs to more effectively monitor their community impact in an efficient, timely, and cost-effective way. Despite that there were minor issues such as the quality of their mobile phone, occasional poor network coverage had little effect on the quality of the data received

**Fig. 3:**
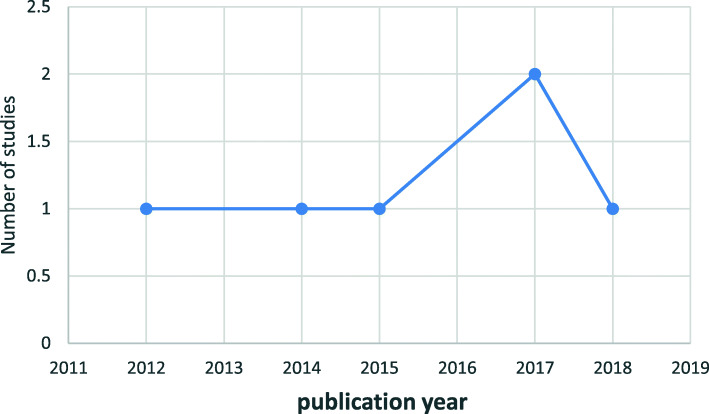
Number of published studies on neglected tropical diseases using electronic technology per year

The included studies in this review were published between 2012 [31] and 2018 [36] (Fig. 3).

### The theme of the study

The primary focus of the included studies fitted broadly into three key themes, namely:

1. Case detection
2. Disease management
3. Treatment outcome

#### Role of digital health technologies for case detection

From all included studies, only one study reported on the use of digital health technology for case detection/identification [33]. Mobile health (mHealth) was deployed for lymphatic filariasis patient identification. The study revealed that the mHealth approach was a feasible framework that can be applied for endemic lymphatic filariasis areas.

#### Digital health technologies for disease management

Out of all included studies, electronic technologies were applied to three studies for disease management purposes. mHealth, eHealth, and electronic health records were for visceral leishmaniasis management [36], and mHealth [35], and mobile message [34] were applied for lymphatic disease management. Experts review by Tambo et al. in Africa [36] revealed that using digital public health technology applications support in improving health service delivery or access and uptake in Africa at the community-health system for managing visceral leishmaniasis. Similarly, a case study in Ghana and Malawi [34] directed that use of mobile messages (SMS) enables trained community-based health workers to report basic information on all cases they identified that enables national programs to more effectively monitor their community impact in an efficient, timely, and cost-effective way. On the other hand, a study in Ghana in 2017 showed that the use of mobile phones is not a guarantee to improve data accuracy, rather the type of mobile technology used may aid in improving data completeness for treatment support [35].

Only one study was used—telehealth for disease diagnosis [31]. An expert review by Bhunia et al. in India directed that telehealth technology can be used for treatment and diagnosis of visceral leishmaniasis through contact with health professionals and teleconsultation.

#### The role of digital health technologies for treatment outcome of neglected tropical disease patients

Only one study revealed the use of electronic technology for neglected tropical disease treatment outcomes. The study was an expert review which showed that the use of mHealth, eHealth, and electronic health records found to improve the service access/delivery and outcomes, monitoring visceral leishmaniasis at the community health system [36].

## Discussion

We conducted a systematic scoping review of studies to explore evidence on electronic technologies used for neglected tropical diseases treatment outcome, case detection, disease management, and disease diagnosis. This study reviewed 10 out of the 20 NTDs identified by the WHO as the most important which have specific drugs for treatment and most commonly found in low- and middle-income countries of Africa, Asia, and Latin America [37].

The findings illustrated that there is limited published research on the use of electronic technologies for neglected tropical diseases treatment outcome, disease management, case detection, and diagnosis. The major findings were reported only for two NTDs (leishmaniasis and lymphatic filariasis) and one reviewed study had information on NTDs in general. In the current review, we observed that there is no increment of studies related to the use of telemedicine and telehealth on NTDs through time. This indicates that there is a need for immediate action from responsible stakeholders to facilitate the application of technologies to reduce the burden of the disease and to achieve a 90% reduction in the number of people requiring interventions against NTDs by 2030, especially for those hard to reach areas [3].

Even though limited studies were reviewed on a few NTDs, the results from reviewed studies demonstrated that the application or intervention of electronic technologies enhances treatment outcome, disease management, case detection, and disease diagnosis of neglected tropical disease for hard to reach and poor health service access areas. Furthermore, other studies conducted on the improvement of disease management and treatment outcome of infectious and chronic disease using electronic technologies [20, 38]. These digital health technologies also have indirect effect by updating timely information, and optimizing transportation network to manage the NTDs. Within the era of digital health, a new source of information such as web searches generated data or social media updates, are emerging as a new promising approach in surveillance, and decision-making support for NTDs [39, 40].

A study done in Africa on leishmaniasis indicates that using mHealth, eHealth, and electronic health records support improving health service access and uptake in Africa at the community health system that able to improve the service delivery and outcomes, and monitoring visceral leishmaniasis [36]. Additionally, a study conducted in India using telehealth on leishmaniasis established that the digital technology facilitates for disease diagnosis, treatment, and vector control. This finding potentially might help to improve medical care in remote rural areas, including many other activities like public awareness, changing personal behavior, early diagnosis and complete treatment, integrated vector management and vector surveillance, social mobilization, and building partnerships [31, 41, 42].

A qualitative study in Tanzania on leishmaniasis, lymphatic filariasis, Chagas disease, and dengue fever in both rural and urban areas revealed that the application of mobile phone-based management information system (MIS) facilitates to control NTDs by quantifying drug need, prioritizing disease at the local level, up warding information for front line health workers in the country. This finding potentially could help treatment outcome and disease management of NTDs for the rest of the African countries and other low- and middle-income countries (LMIC) [43, 44]. Similarly, a study in Ghana and Malawi using mobile message (SMS) on lymphatic filariasis established that using a developed SMS tool enables trained community-based health workers to report basic information on all cases they identified [34]. On the other hand, the finding revealed that there are some problems such as quality of personal mobile phone and occasional poor network coverage that affects the quality data received.

### Limitation of the review

Including studies conducted in both rural and urban settings which provide an overview of digital health technologies on case detection, disease management, and treatment outcome of neglected tropical diseases in the world—the strength of the study. To the best of our knowledge, this is the first comprehensive review in the world to see the sights of electronic technologies to improve NTDs treatment outcome, case detection, disease diagnosis, and management. The other strength of this review was incorporating any types of study design, and expertise review as inclusion criteria and removal of limitation of language and date studies. However, our review has the following limitations: Scoping reviews are broad and provide an overview of existing literature regardless of quality, providing a broader and more contextual overview than systematic reviews. A formal assessment of methodological quality is neither undertaken when conducting a scoping review and synthesis of the incorporating studies nor is the demonstration of a cause and effect nature for the found relationships not possible due to the nature of the included study were another possible limitation.

## Conclusion

This scoping review found that there were limitations of studies on NTDs intervention using digital health technologies that increase the overall success of the NTD control program in low- and middle-income countries especially areas hard to reach and poor availability of health service delivery. The review also found that digital health technologies were applied to limited numbers of NTDs in a very small number of countries.

The current scoping review showed that using digital public health technology applications like mHealth, eHealth, electronic medical linkage, telemed, and telehealth were feasible for patient identification, for treatment and diagnosis through contact with health professionals and teleconsultation, and support in improving health service delivery at the community-health system for managing the disease.

### Recommendation for future research

This review study revealed that there are limited published researches on the use of electronic technologies for treatment outcome, disease management, case finding, and detection of neglected tropical diseases. Even the limited published researches were conducted on very few NTDs. Therefore, we recommended that more primary studies could be conducted in all types of NTDs to get more evidence and to examine the use of electronic technologies for treatment, management case finding, and detection. The review also showed that mobile phone messages/reminders, electronic medical records, and eHealth were used to improve disease management and treatment outcome. Finally, we recommended that experimental study designs using different digital health technologies for case detections, disease management, and treatment outcome of NTDs could be conducted.

## Supplementary Information

**Additional file 1:.** Keywords included in the search strategy for PubMed databases; terms searched for in the title and abstract of papers.

**Additional file 2:.** Extracted data.

## Data Availability

The datasets supporting the conclusions of this article are included in the review.

## Abbreviations

JBI: Joanna Briggs Institute
NTD: Neglected tropical diseases
PRISMA-ScR: Preferred Reporting Items for Systematic reviews and Meta-Analyses extension for Scoping Reviews
WHO: World health organization
