# Supplementary material for: Mapping the role of digital health technologies in the case detection, management, and treatment outcomes of neglected tropical diseases: a scoping review
Source: Trop Med Health. 2021 Feb 22;49:17. doi: 10.1186/s41182-021-00307-1 (PMC7898439; doi:10.1186/s41182-021-00307-1)
Supplement: Supplementary file 1 — Additional file 1:. Keywords included in the search strategy for PubMed databases; terms searched for in the title and abstract of papers. [file 41182_2021_307_MOESM1_ESM.docx]

Table 1 Keywords included in the search strategy for PubMed databases; terms searched for in the title and abstract of papers

| **Mesh Heading** | **Entry terms** | **Searching combination** | Searching date | Total records |
| --- | --- | --- | --- | --- |
| **neglected tropical disease** |  |  |  |  |
| Leishmaniasis | Leishmania Infection,visceral Leishmania,cutaneous Leishmania, mucocutaneous Leishmania  kalazar | **((telemed OR MobileHealth OR Telehealth OR eHealth OR mHealth OR "Computerized Medical Record*" OR "Electronic Health Record " OR "Electronic Medical Record" OR (Medical Record, Computerize) OR Record Linkage, Medical) AND (leishmaniasis[Title/Abstract] OR Leishmania Infection[Title/Abstract] OR visceral Leishmania[Title/Abstract] OR cutaneous Leishmania[Title/Abstract] OR mucocutaneous Leishmania[Title/Abstract])) AND (treatment outcome[Title/Abstract] OR "Clinical Effectiveness"[Title/Abstract] OR " Clinical Efficacy Patient-Relevant Outcome"[Title/Abstract] OR "Rehabilitation Outcome"[Title/Abstract] OR "Treatment Effectiveness"[Title/Abstract] OR "Treatment Efficacy"[Title/Abstract] OR lost to follow-up[Title/Abstract] OR "Dis management"[Title/Abstract] OR "Managed Care Programs" [Title/Abstract] OR "Patient Care Planning"[Title/Abstract] OR " disease management"[Title/Abstract])** | **29/07/2020** | **56** |
| Schistosmiasis | Bilharziasis  Katayama Fever  Schistoma Infectio  Schistoma mansoni  Schistoma japonicum  Schistoma hematobium  Snail fever | **(((telemed OR MobileHealth OR Telehealth OR eHealth OR mHealth OR "Computerized Medical Record*" OR "Electronic Health Record " OR "Electronic Medical Record" OR (Medical Record, Computerize) AND (Schistosomiasis OR "Bilharziasis Katayama" OR "Fever Schistoma" OR " Infection Schistoma"[Title/Abstract] OR mansoni OR "Schistoma japonicum" OR “**Schistoma hematobium”**)) AND (treatment outcome OR "Clinical Effectiveness" OR " Clinical Efficacy Patient-Relevant Outcome" OR "Rehabilitation Outcome" OR "Treatment Effectiveness" OR "Treatment Efficacy" OR lost to follow-up OR "Dis management" OR "Managed Care Programs" OR "Patient Care Planning" OR " disease management" OR " case finding" OR "case detection")** | **29/07/2020** | **396** |
| Elephantiasis, Filarial | Bancroftian Elephantiasis  Bancroftian Filariasis  Elephantiasis,  Bancroftian Elephantiasis,  Malayi Filarial  Elephantiasis Filariasis,  Lymphatic Filariasis  Malayi Filariasis | **((OR "Bancroftian Elephantiasis"[Title/Abstract] OR "Bancroftian Filariasis"[Title/Abstract] OR Elephantiasis[Title/Abstract] OR "Bancroftian Elephantiasis"[Title/Abstract] OR "Malayi Filarial"[Title/Abstract] OR "Elephantiasis Filariasis"[Title/Abstract] OR " Lymphatic Filariasis"[Title/Abstract] OR " Malayi Filariasis"[Title/Abstract]) AND (treatment outcome OR "Clinical Effectiveness" OR " Clinical Efficacy Patient-Relevant Outcome" OR "Rehabilitation Outcome" OR "Treatment Effectiveness" OR "Treatment Efficacy" OR lost to follow-up OR "Dis management" OR "Managed Care Programs" OR "Patient Care Planning" OR " disease management" OR " case finding" OR "case detection")) AND (telemed OR MobileHealth OR Telehealth OR eHealth OR mHealth OR "Computerized Medical Record*" OR "Electronic Health Record " OR "Electronic Medical Record" OR (Medical Record, Computerize) OR Record Linkage, Medical)** | **01/08/2020** | **3** |
| Trachoma | Egyptian Ophthalmia,trichiasis,clamidial infectiobn,truh-koh-nuh | **((Trachoma[Title/Abstract] OR "Egyptian Ophthalmia"[Title/Abstract] OR trichiasis[Title/Abstract] OR "clamidial infection"[Title/Abstract] OR truh-koh-nuh[Title/Abstract]) AND (treatment outcome OR "Clinical Effectiveness" OR " Clinical Efficacy Patient-Relevant Outcome" OR "Rehabilitation Outcome" OR "Treatment Effectiveness" OR "Treatment Efficacy" OR lost to follow-up OR "Dis management" OR "Managed Care Programs" OR "Patient Care Planning" OR " disease management" OR " case finding" OR "case detection")) AND (telemed[Title/Abstract] OR MobileHealth[Title/Abstract] OR Telehealth[Title/Abstract] OR eHealth[Title/Abstract] OR mHealth[Title/Abstract] OR "Computerized Medical Record*"[Title/Abstract] OR "Electronic Health Record "[Title/Abstract] OR "Electronic Medical Record"[Title/Abstract] OR (Medical Record, Computerize)[Title/Abstract] OR Record Linkage, Medical[Title/Abstract])** | **01/08/2020** | **72** |
| Dracunculiasis | Dracunculosis  Guinea Worm Disease  Guinea Worm Infection | **((Dracunculiasis[Title/Abstract] OR Dracunculosis[Title/Abstract] OR Guinea Worm Disease[Title/Abstract] OR "Guinea Worm Infection"[Title/Abstract]) AND (treatment outcome OR "Clinical Effectiveness" OR " Clinical Efficacy Patient-Relevant Outcome" OR "Rehabilitation Outcome" OR "Treatment Effectiveness" OR "Treatment Efficacy" OR lost to follow-up OR "Dis management" OR "Managed Care Programs" OR "Patient Care Planning" OR " disease management" OR " case finding" OR "case detection")) AND (telemed OR MobileHealth OR Telehealth OR eHealth OR mHealth OR "Computerized Medical Record*" OR "Electronic Health Record " OR "Electronic Medical Record" OR (Medical Record, Computerize) OR Record Linkage, Medical)** | **01/08/2020** | **13** |
| Soil-transmitted Helminths | **Ascariasis**  **Hookwarm**  **whipworm** | **((Soil-transmitted Helminths [Title/Abstract] ascariasis OR hookworm OR whipworm [Title/Abstract]) AND (treatment outcome OR "Clinical Effectiveness" OR " Clinical Efficacy Patient-Relevant Outcome" OR "Rehabilitation Outcome" OR "Treatment Effectiveness" OR "Treatment Efficacy" OR lost to follow-up OR "Dis management" OR "Managed Care Programs" OR "Patient Care Planning" OR " disease management" OR " case finding" OR "case detection")) AND (telemed OR MobileHealth OR Telehealth OR eHealth OR mHealth OR "Computerized Medical Record*" OR "Electronic Health Record " OR "Electronic Medical Record" OR (Medical Record, Computerize) OR Record Linkage, Medical)** | **01/08/2020** | **60** |
| [Onchocerciasis](https://www.cdc.gov/parasites/onchocerciasis/index.html) | **River blindness** | **((Onchocerciasis[Title/Abstract]) OR river blindness [Title/Abstract]) AND (treatment outcome OR "Clinical Effectiveness" OR " Clinical Efficacy Patient-Relevant Outcome" OR "Rehabilitation Outcome" OR "Treatment Effectiveness" OR "Treatment Efficacy" OR lost to follow-up OR "Dis management" OR "Managed Care Programs" OR "Patient Care Planning" OR " disease management" OR " case finding" OR "case detection")) AND (telemed OR MobileHealth OR Telehealth OR eHealth OR mHealth OR "Computerized Medical Record*" OR "Electronic Health Record " OR "Electronic Medical Record" OR (Medical Record, Computerize) OR Record Linkage, Medical)** | **02/08/2020** | **103** |
| [Chagas Disease](https://www.cdc.gov/parasites/chagas) | American Trypanosomiasis  Chagas' Disease  Trypanosoma cruzi Infection  Trypanosomiasis | **(("Chagas Disease"[Title/Abstract] OR "American Trypanosomiasis" [Title/Abstract] OR "Chagas' Disease"[Title/Abstract] OR " Trypanosoma cruzi Infection "Trypanosomiasis[Title/Abstract]) AND (treatment outcome OR "Clinical Effectiveness" OR " Clinical Efficacy Patient-Relevant Outcome" OR "Rehabilitation Outcome" OR "Treatment Effectiveness" OR "Treatment Efficacy" OR lost to follow-up OR "Dis management" OR "Managed Care Programs" OR "Patient Care Planning" OR " disease management" OR " case finding" OR "case detection")) AND (telemed OR MobileHealth OR Telehealth OR eHealth OR mHealth OR "Computerized Medical Record*" OR "Electronic Health Record " OR "Electronic Medical Record" OR (Medical Record, Computerize) OR Record Linkage, Medical)** | **02/08/2020** | **22** |
| Dengue | Break-Bone Fever  Breakbone Fever  Classical Dengue  Classical Dengue Fever  Dengue Fever | **((telemed[Title/Abstract] OR MobileHealth[Title/Abstract] OR Telehealth[Title/Abstract] OR eHealth[Title/Abstract] OR mHealth[Title/Abstract] OR "Computerized Medical Record*"[Title/Abstract] OR "Electronic Health Record "[Title/Abstract] OR "Electronic Medical Record"[Title/Abstract] OR (Medical Record, Computerize)[Title/Abstract] OR Record Linkage, Medical[Title/Abstract]) AND (treatment outcome OR "Clinical Effectiveness" OR " Clinical Efficacy Patient-Relevant Outcome" OR "Rehabilitation Outcome" OR "Treatment Effectiveness" OR "Treatment Efficacy" OR lost to follow-up OR "Dis management" OR "Managed Care Programs" OR "Patient Care Planning" OR " disease management" OR " case finding" OR "case detection")) AND (dengue OR "Break-Bone Fever" OR " Breakbone Fever" OR " Classical Dengue" OR " Classical Dengue Fever" OR "Dengue Fever"")** | **02/08/2020** | **1** |
| [Human African Trypanosomiasis](https://www.cdc.gov/parasites/sleepingsickness) | **Sleeping sickness** | **((telemed[Title/Abstract] OR MobileHealth[Title/Abstract] OR Telehealth[Title/Abstract] OR eHealth[Title/Abstract] OR mHealth[Title/Abstract] OR "Computerized Medical Record*"[Title/Abstract] OR "Electronic Health Record "[Title/Abstract] OR "Electronic Medical Record"[Title/Abstract] OR (Medical Record, Computerize)[Title/Abstract] OR Record Linkage, Medical[Title/Abstract]) AND (treatment outcome OR "Clinical Effectiveness" OR " Clinical Efficacy Patient-Relevant Outcome" OR "Rehabilitation Outcome" OR "Treatment Effectiveness" OR "Treatment Efficacy" OR lost to follow-up OR "Dis management" OR "Managed Care Programs" OR "Patient Care Planning" OR " disease management" OR " case finding" OR "case detection")) AND ("Human African Trypanosomiasis"[Title/Abstract])** | **02/08/2020** | **64** |
| **MeSH heading** | **Entry term** | | | |
| **Treatment outcome** | - Clinical Effectiveness - Clinical Efficacy - Patient-Relevant Outcome - Rehabilitation Outcome - Treatment Effectiveness - Treatment Efficacy | | | |
| Lost to follow up | - **Attrition** | | | |
| Disease management | - **Dis** management - Managed Care Programs - Patient Care Planning | | | |
| **Electronics technology** |  | | | |
| Telemedicine | - Telemed - MobileHealth - Telehealth - eHealth, - mHealth | | | |
| Mobile message/reminder | - **Short message service(SMS)** | | | |
| Electronic medical record | - Computerized Medical Record - Computerized Medical Records - Electronic Health Record - Electronic Medical Record - Electronic Medical Records - Medical Record, Computerized - Medical Records, Computerized | | | |
| Electronic health records | - Computerized Medical Record - Computerized Medical Records - Electronic Health Record - Electronic Medical Record - Electronic Medical Records - Medical Record, Computerized - Medical Records, Computerized | | | |
| Medical Record Linkage | - Record Linkage, Medical | | | |
